# Minimally invasive management of a giant paratubal cyst in an adolescent female: Case report and review of the literature in the pediatric population

**DOI:** 10.3389/fped.2022.1080797

**Published:** 2022-12-07

**Authors:** Paola Romeo, Giada Loria, Canio Martinelli, Alfredo Ercoli, Carmelo Romeo

**Affiliations:** ^1^ Department of Human Pathology of Adult and Childhood “Gaetano Barresi”, Unit of Obstetrics and Gynecology, University of Messina, Messina, Italy; ^2^ Department of Human Pathology of Adult and Childhood “Gaetano Barresi”, Unit of Pediatric Surgery, University of Messina, Messina, Italy

**Keywords:** minimally invasive surgery, giant paratubal cyst, paraovarian cyst, laparoscopy, case report

## Abstract

**Introduction:**

Paraovarian or paratubal cysts both define cysts located between the ovary and the fallopian tube. They are usually benign and frequently occur in the third and fourth decade of life. Paratubal cysts are defined as giant when they exceed the threshold of 150 mm.

**Methods:**

We report the case of a 15-year-old girl who complained about diffuse abdominal pain since 2 years that was diagnosed with a 196 mm × 90 mm × 267 mm giant paratubal cyst. We furthermore reviewed all the data published on 13 articles, published between 2006 and 2021, concerning giant paraovarian cyst (POC) in pediatric patients.

**Results:**

The giant mass of our 15-year-old patient was removed through a fertility-sparing laparoscopic surgery. Histopathological diagnosis of cystadenofibroma was made up, with no cytologic report of neoplastic cells. The incidence of POC in the pediatric and adolescent population attests around 4%. However, only 12.96% of them are defined giant (larger than 15 cm). Indeed, to the best of our knowledge, only 13 cases of giant paratubal cysts have been reported in adolescents. To accomplish diagnosis and differential diagnosis, accurate history and physical examination are mandatory. In all cases reported in the literature, further instrumental analyses were performed, including ultrasound, CT, and/or MRI scan. International Ovarian Tumor Analysis (IOTA) rules have not yet been validated in the pediatric population. Because of the advantages of the laparoscopic procedures, it is often preferred in pediatric population, also to help preserve as much ovarian parenchyma and salpinx if thought possible. The incidence of malignant adnexal masses in the pediatric population is reported to range from 4% to 9%, accounting for 1% of all pediatric cancers.

**Conclusion:**

Giant paratubal cysts in adolescent females are extremely rare and usually benign. A fertility-sparing laparoscopic surgery should be the preferable option whenever possible. Considering the rarity of these conditions, further investigations are needed to exclude the possibility of a malignant evolution.

## Introduction

Paraovarian or paratubal cysts are terms that can be used interchangeably: they both define cysts located between the ovary and the fallopian tube and they make up about 10% of all adnexal masses (1, 2). These cysts are usually benign and originate most commonly from the mesothelium. Moreover, they can also be remnants of the paramesonephric (Mullerian) and mesonephric (Wolffian) ducts. The peak age of occurrence is in the third and fourth decades of life (3). In particular, 4% of paratubal cysts occur in adolescence (4, 5), whereas their prevalence in postmenopausal women seems to be of 6.25% (6). The mean size of paratubal cysts has been reported to be 75.1 mm (7). It is not clear when a cyst should be considered “giant”: some authors consider that the size must exceed the threshold of 150 mm (8, 9) and just 12.96% of paratubal cysts exceed that size making giant paratubal cysts quite rare (10).

Only a few cases of giant paratubal cysts have been published, particularly as far as the pediatric population is concerned. Moreover, all these cases had different surgical approaches. Therefore, our aim is to report our case of a giant paratubal cyst (maximal diameter of 26.7 cm) in a 15-year-old patient that was managed by minimally invasive surgery and to report a review of the literature on giant paratubal cysts in the pediatric population.

## Case presentation

**Figure 1:**
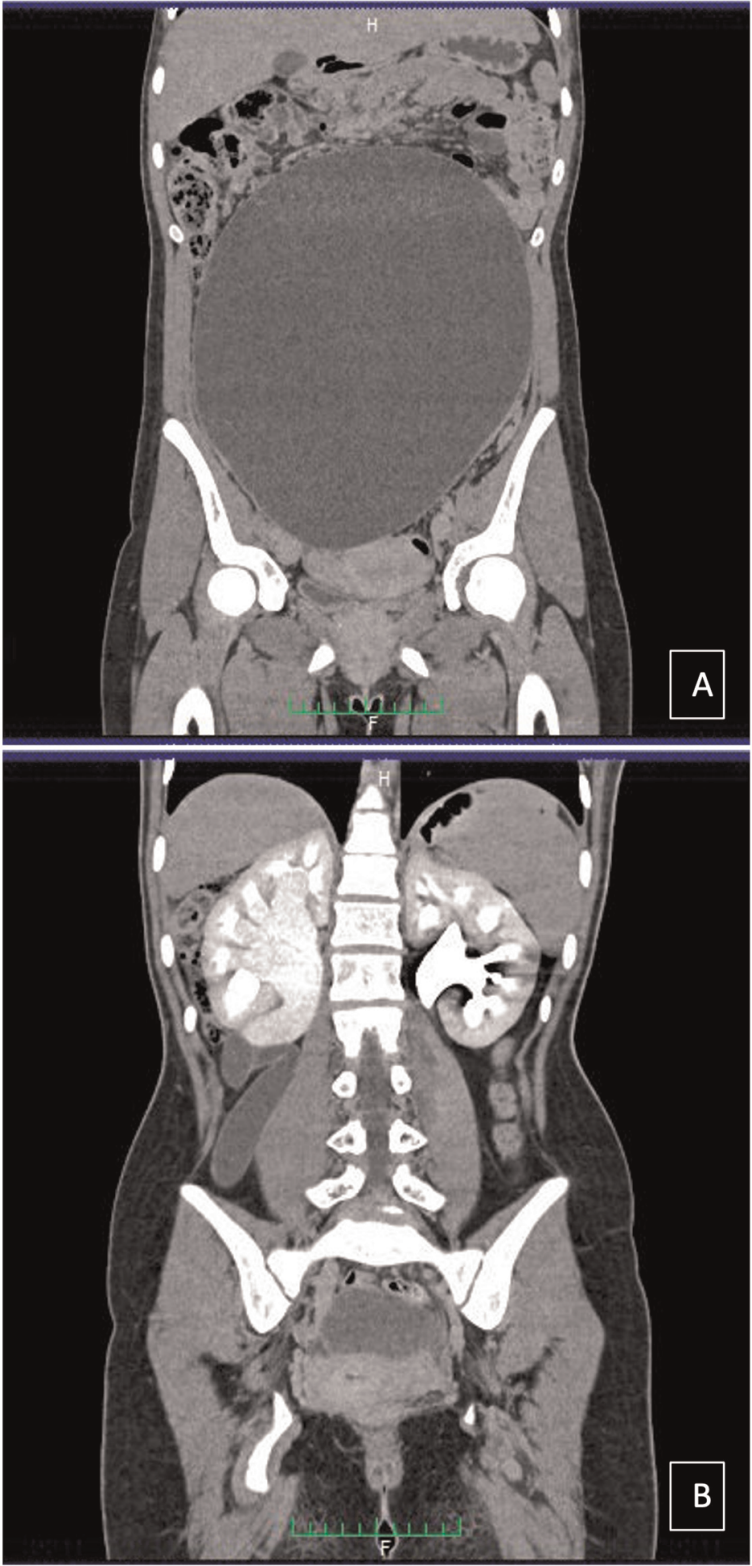
Computed tomography scan of the of giant paratubal cyst of 196 mm × 90 mm × 267 mm, coming from the pelvis and occupying all the four abdominal quadrants (**A**) and causing a IV grade hydronephrosis on the right kidney (**B**).

**Figure 2:**
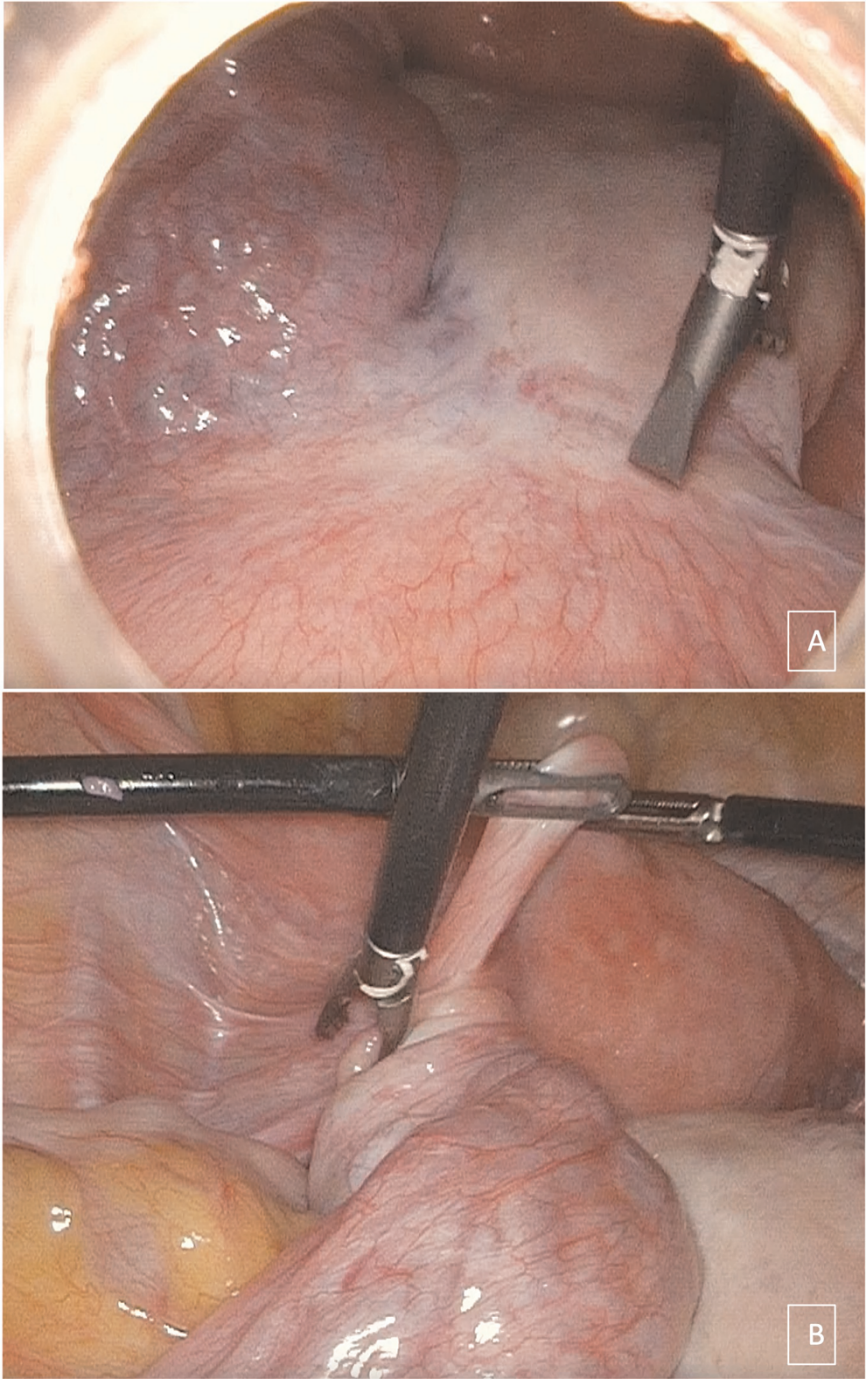
Laparoscopic appearance of part of the giant paratubal cyst (**A**) and of the convoluted left tube (**B**).

**Figure 3:**
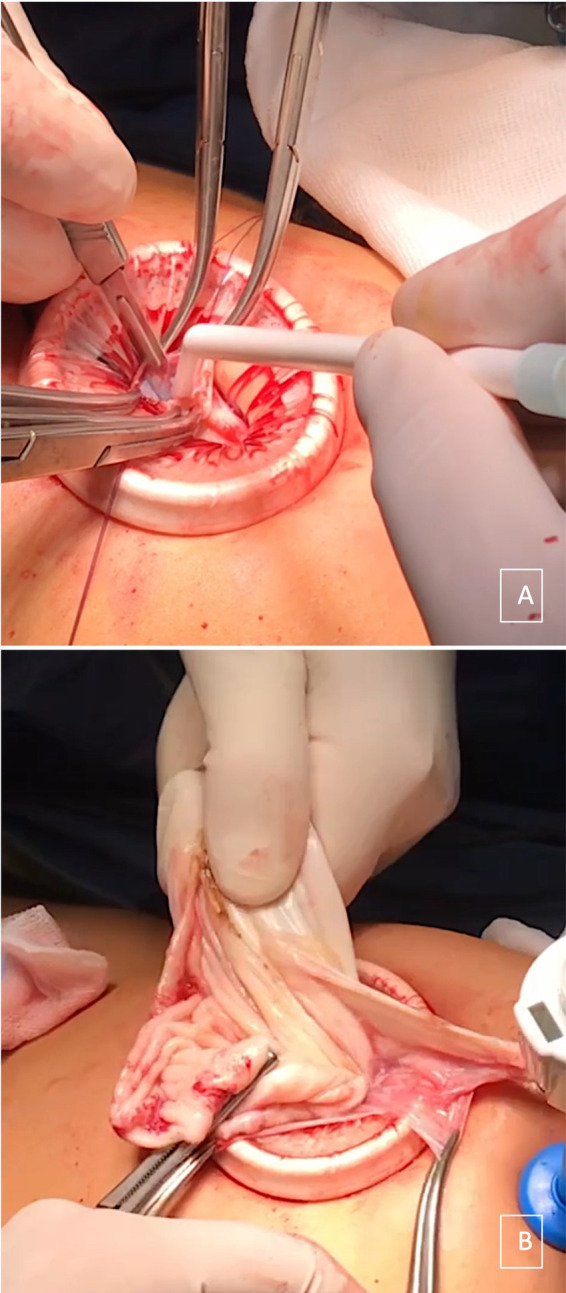
Alexis XS retractor inserted at the level of the umbilical access in order to carefully puncture the cyst using a suction irrigation apparatus (**A**), and extracorporeal cyst excision (**B**).

A 15-year-old girl came to our attention because of diffuse abdominal pain. She was virgo, her medical history was uneventful, and she reported regular menstrual cycles. Family history and medical history were irrelevant. The patient complained about abdominal distension for 2 years. No other symptoms were reported apart from vomiting that started in the last few days. At physical examination, a firm and painless abdominal mass extending from the pubis to 2–3 cm above the umbilicus was evident. We performed a transabdominal ultrasound examination that showed a unilocular mass occupying the whole abdomen until the epigastrium. The mass was 300 mm × 240 mm × 120 mm in size, with regular walls and anechoic content and no vascularization at Color Doppler examination was observed. Free fluid was present in the Pouch of Douglas. Serum levels of oncological markers were all negative: Ca125: 12.2 U/ml, Ca19.9: 5.7 U/ml, and Ca15.3: 19.6 U/ml. In order to better characterize the mass, the patient underwent abdominal computed tomography (CT) scan that reported a voluminous endoabdominal lesion of 196 mm × 90 mm × 267 mm coming from the pelvis and occupying all the four abdominal quadrants (Figure 1A). The cyst had regular walls, fluid-type density, and absence of solid components. The mass was described as hardly dissociable from the adnexa from where it seemed to come from. Moreover, it caused renal, bladder, and urinary tract compression causing a grade IV hydronephrosis on the right kidney (Figure 1B). At the level of the median presacral area, another cystic neoformation, with fluid content and a diameter of 60 mm × 69 mm, was observed. The presence of free fluid in the Pouch of Douglas was confirmed with CT scan too. At this point, the patient was admitted, and the differential diagnosis included ovarian lesion, either benign or malignant, tubal or paratubal lesion, tubo-ovarian abscess, and nongynecologic disease, such as peritoneal inclusion cysts or appendiceal abscess. In our opinion, the cyst had benign morphology. However, in order to confirm the origin and characteristics of the cyst, an exploratory surgery performed by pediatric surgeons together with gynecologists was planned. To be as least invasive as possible, a transumbilical laparoscopy (LPS) was performed. At inspection, a left paraovarian lesion occupying the whole pelvis until the transpyloric plane was detected (Figure 2A). The left tube showed multiple torsions around its axis causing lymphatic and vascular congestion (Figure 2B). The left ovary was regular in morphology with slightly increased size. The uterus showed regular size and morphology. The right adnexa was normal and a Morgagni hydatid cyst was detected. Free fluid was present. Three operative trocars were inserted, and the free fluid was aspirated and sent for definitive cytological examination. Because of the huge dimension of the cyst, the pneumoperitoneum was resolved, the optical trocar was removed, and an Alexis XS retractor was inserted in order to exteriorize and incise the cyst at the level of the umbilical access. The cyst was carefully punctured and using a suction irrigation apparatus, a total of 2800 cc of fluid was aspirated from the cyst with no spillage (Figure 3A). At this point, we performed extracorporeal cyst excision (Figure 3B). After that, a careful hemostasis was obtained, the optical trocar was reinserted, and pneumoperitoneum was reinduced. Left tubal detorsion was now possible. Fertility-sparing surgery was our main goal; however, because of the ischemic appearance of the tube, we proceeded to a laparoscopic right salpingectomy with homolateral ovary conservation. The patient had an uneventful clinical course and was discharged after 3 days. Cytology was negative for tumor cells and final histology report confirmed a cystadenofibroma with cuboidal monostratified walls. The tube showed signs of hyperemia and peritubal congestion. At 1-week follow,-up transabdominal ultrasound was normal and hydronephrosis was resolving (grade II hydronephrosis). At 6-month follow-up, the patient was doing well and hydronephrosis had completely resolved.

The patient's parents provided consent for publication of this case report.

## Discussion and review of the literature

Paratubal cysts make up about 10% of all adnexal masses (1, 2). The incidence of paratubal cysts in the pediatric and adolescent population seems to be of 4% (4, 5). However, only 12.96% of paratubal cysts are larger than 15 cm (giant cysts) (10). Indeed, to the best of our knowledge, only 13 cases of giant paratubal cysts have been reported in adolescents (Table 1). The size of the giant cysts in adolescents reported in the literature ranged from 17 to 40 cm. The differential diagnosis of adnexal masses in pediatric and adolescent females is broad, including ovarian lesions, either benign or malignant, tubal or paratubal lesions, pelvic inflammatory disease, tubo-ovarian abscess, and nongynecological disease, such as peritoneal inclusion cysts or appendiceal abscess (11). Thus, an accurate history and physical examination are mandatory. In all cases reported in the literature, ultrasound scan was used in order to diagnose the mass, even if it was always quite impossible differentiating between ovarian and paraovarian lesion. Indeed, the primary value of ultrasound is the possibility it offers to distinguish simple unilocular cysts, with no solid components, absence of blood flow and regular margins, which are usually benign, from solid masses, with irregular margins, and abundant blood flow, that are usually malignant. In addition to that, ultrasound imaging can assist in discriminating adnexal masses from gastrointestinal and uterine pathology (11). According to some studies (12), the risk of malignancy of paratubal cysts is higher when the size of the cyst is bigger than 5 cm, but other differentiating criteria were not investigated. For this reason, no other criteria apart from International Ovarian Tumor Analysis (IOTA) simple rules, which have a sensitivity of 95% and a specificity of 91% in discriminating between benign and malignant adnexal masses, are available (13). However, IOTA rules have not yet been validated in the pediatric population. Hence, in order to establish a proper diagnosis, CT or MRI imaging are almost always needed. Indeed, in most cases (7/13) of giant paratubal cyst reported in the literature, CT scan was performed to clarify the diagnosis. However, in the pediatric population, we should also consider the risk of radiation exposure and damage to the ovaries. Indeed, a study published in 2013 demonstrated that among 680,000 patients who underwent CT scans under the age of 19 years, the overall cancer incidence in the next 10 years was 24% higher in patients who had undergone CT scans vs. those who had not. Even if CT scan radiation doses were likely higher in the study period than they are now, some increase in cancer risk is still likely (14). For this reason, in case of diagnostic doubts, magnetic resonance imaging should be preferred. Nevertheless, despite all the imaging diagnostic efforts, the exact origin of such voluminous cysts is most often revealed only by surgical exploration. For this reason, a close collaboration between gynecologist and pediatric surgeon should be achieved, because the presence of both may be essential at the operating theater.

**Table 1:** Review of cases of giant paratubal cysts in the pediatric population.

Authors, year	Age	Size (mm)	Imaging methods	Surgical management	Histology
Mukhopadhyay, 2006	18	400	US	CE, SO	Simple serous cyst
Kostov et al., 2008	14	300	US	AE	Serous cystadenoma
Saxena et al., 2008	16	300	US, MRI	LCE	Serous cystadenoma
Kandil et al., 2013	17	260	CT	CE	Simple serous cyst
Leanza et al., 2013	14	300	US	LCE	Paramesonephric cyst
Asare et al., 2015	19	270	US, CT	CE	Paratubal cyst
Erikci et al., 2015	14	400	US, CT	CE, SE	Serous cystadenoma
Shah et al., 2016	16	260	US, CT	LCE	Paratubal cyst
Lee et al., 2016	17	190	US, CT	LCE, SE	Serous papillary cystadenoma
Marginean et al., 2018	15	170	US, MRI	LCE	Serous cyst
Zvizdic et al., 2020	15	250	US, MRI	CE	Serous cystadenoma
Kiran et al., 2021	13	230	US, CT	LCE	Serous cystadenoma
Torres et al., 2015	13	200	US, CT	LCE	Paraovarian cyst

US, ultrasonography; CT, computed tomography; MRI, magnetic resonance imaging; SO, salpingo-oophorectomy; LCE, laparoscopic cystectomy; SE, salpingectomy; CE, cystectomy; AE, adnexectomy.

The management of the cysts should be based on the imaging characteristics and the clinical appearance. Traditionally, giant paratubal cysts have been managed with midline laparotomy (15). However, because of the advantages of the laparoscopic procedures such as better patient recovery, shorter hospital stay, less postoperative pain, and a better magnification of the image (16) that may be essential in the pediatric population to help in preserving as much ovarian parenchyma and salpinx as possible, the minimally invasive approach is nowadays being preferred for giant paratubal cysts. However, according to our review, laparoscopy was possible in 7/13 of adolescents, whereas laparotomy was preferred in the other cases. These results are in accordance with a very recent review on the management of paratubal cyst in the general population, in which minimally invasive techniques were applied in only 42.9% of cases despite its advantages (17). This may be explained by the fact that in order to manage cysts of this size, a high-level laparoscopic expertise is needed. In our case, we were able to perform a laparoscopic-assisted surgery with the help of an Alexis XS retractor that was inserted at the level of the umbilical access to puncture the cyst using a suction irrigation apparatus. After careful fluid aspiration, extracorporeal cyst excision was achieved, and the cyst was sent for final histology examination. We did not perform any intraoperative frozen section, and we assume that it should not be performed. Indeed, a recent meta-analysis concluded that in a hypothetical population of 1,000 patients with ovarian disease, if a frozen section result of either invasive tumor or borderline tumor was used to diagnose cancer, 280 women would be correctly diagnosed with cancer and 635 women without cancer; however, 75 women would be incorrectly diagnosed with cancer and 10 would be missed (18). Hence, we cannot risk performing an adnexectomy in a pediatric patient if we are not almost 100% sure it is a cancer. For this reason, final histology report should be the only indication.

It is important to highlight that, regardless of the approach employed, fertility preservation should be prioritized when possible. According to that, it seems that surgeon specialty and surgical outcome are linked. Indeed, Eskander and Bristow found out in a retrospective review of 82 cases that the presence of a gynecologic surgeon was significantly and independently associated with ovarian preservation (OR: 8.71; 95% CI: 2.12–41.41; *P* = 0.001) (19). This further confirms the importance of a multidisciplinary work-up. According to our review, fertility-sparing surgery has been achieved in 11/13 adolescents presenting with giant paratubal cysts (in 2 of them, cystectomy and homolateral salpingectomy were performed), whereas homolateral adnexectomy was performed in 2/13 cases. In our case, we were not able to preserve the homolateral salpinx because of the ischemic appearance of the tube that was twisted around its axis. In relation to that, a very recent review of giant paratubal cysts in the general population reported that ovarian-sparing surgery was performed in 85.1% of cases; however, according to them, the risk of torsion is very low (11.1%), thus making tubal sparing achievable (17).

Regarding the final histology of the removed cyst, the final histology report of the reviewed cases was of serous cystadenoma in 13/13 cases. Final diagnosis of our cyst was cystadenofibroma with cuboidal monostratified walls. It is important to emphasize that, even if in the general population paratubal cysts have almost always a benign nature, an unexpected high percent (25.9%) of borderline giant paratubal cysts has been found (17). Moreover, regarding the incidence of malignant adnexal masses in pediatric and adolescent population, it is reported to range from 4% to 9%, accounting for 1% of all pediatric cancers (20). The most common ovarian neoplasm is germ cell tumor (from 67% to 85%), and nearly one-third of them are malignant (21). Dysgerminomas are the most common malignant germ cell tumors (30%–40% of all ovarian germ cell tumors). After them, in order of decreasing frequency, we can find immature teratomas, endodermal sinus tumors, embryonal carcinomas, choriocarcinomas, and polyembryomas. On the other hand, sex cord–stromal tumors account for 5%–8% of all ovarian malignancies (11). Hence, even if no cases of borderline or malignant tumors have been found in giant paratubal cysts in the pediatric population, a higher awareness of the possibility of these types of histology is required for the proper management of these peculiar cases.

In conclusion, giant paratubal cysts in adolescent females are extremely rare, and they usually have an excellent prognosis. However, because of the complexity and rarity of these conditions, for proper management, patients should be treated at a tertiary referral center by an experienced multidisciplinary team. Laparoscopy should be preferred when performed by an experienced surgeon, and a fertility-sparing surgery should be the preferable option whenever possible. Better knowledge on the possibility of a borderline or malignant histology in giant paratubal cysts is required.

## Data Availability

The original contributions presented in the study are included in the article/Supplementary Material, further inquiries can be directed to the corresponding author.
